# A global analysis of CNVs in diverse yak populations using whole-genome resequencing

**DOI:** 10.1186/s12864-019-5451-5

**Published:** 2019-01-18

**Authors:** Hui Wang, Zhixin Chai, Dan Hu, Qiumei Ji, Jinwei Xin, Chengfu Zhang, Jincheng Zhong

**Affiliations:** 1 Key Laboratory of Qinghai-Tibetan Plateau Animal Genetic Resource Reservation and Utilization (Southwest Minzu University), Ministry of Education, Chengdu, 610000 People’s Republic of China; 2 State Key Laboratory of Barley and Yak Germplasm Resources and Genetic Improvement, Tibet Academy of Agricultural and Animal Husbandry Sciences, Lhasa, 850000 People’s Republic of China

**Keywords:** *Bos grunniens*, CNV, Environmental adaptation, Cluster analysis

## Abstract

**Background:**

Genomic structural variation represents a source for genetic and phenotypic variation, which may be subject to selection during the environmental adaptation and population differentiation. Here, we described a genome-wide analysis of copy number variations (CNVs) in 16 populations of yak based on genome resequencing data and CNV-based cluster analyses of these populations.

**Results:**

In total, we identified 51,461 CNV events and defined 3174 copy number variation regions (CNVRs) that covered 163.8 Mb (6.2%) of yak genome with more “loss” events than both “gain” and “both” events, and we confirmed 31 CNVRs in 36 selected yaks using quantitative PCR. Of the total 163.8 Mb CNVR coverage, a 10.8 Mb region of high-confidence CNVRs directly overlapped with the 52.9 Mb of segmental duplications, and we confirmed their uneven distributions across chromosomes. Furthermore, functional annotation indicated that the CNVR-harbored genes have a considerable variety of molecular functions, including immune response, glucose metabolism, and sensory perception. Notably, some of the identified CNVR-harbored genes associated with adaptation to hypoxia (e.g., *DCC, MRPS28*, *GSTCD*, *MOGAT2*, *DEXI*, *CIITA*, and *SMYD1*). Additionally, cluster analysis, based on either individuals or populations, showed that the CNV clustering was divided into two origins, indicating that some yak CNVs are likely to arisen independently in different populations and contribute to population difference.

**Conclusions:**

Collectively, the results of the present study advanced our understanding of CNV as an important type of genomic structural variation in yak, and provide a useful genomic resource to facilitate further research on yak evolution and breeding.

**Electronic supplementary material:**

The online version of this article (10.1186/s12864-019-5451-5) contains supplementary material, which is available to authorized users.

## Background

Copy number variation (CNV), defined as deletion or duplication of DNA fragments larger than 50 bp in length compared with a reference genome, is a ubiquitous form of genomic structural variation [1, 2], and has pronounced effects on phenotype [3, 4] and evolutionary adaptation [5, 6] through altering gene expression levels or transcript structure. Many previous publications have reported the effects of CNVs on evolution [7], population diversity [8, 9], and various physiological processes(e.g., lipid metabolism [4, 10] and glucose metabolism [11]) and pathological processes (e.g., cancer related biologic processes [12] and the occurrence and progression of many tumors [13]). Furthermore, CNV provides the mechanisms and resources for creating new genes [14].

Recent studies on the distribution of CNVs in the human genome have shown that more than 12% of the human genome containing CNVs [15]. Given the ubiquitous distribution of CNVs and their importance, advances in CNV detection have extended to livestock and poultry species, including pigs [16], goats [17], sheep [18], cattle [19], and chicken [20]. These animal datasets provide a very valuable resource for evolution and genetic improvement research. Interestingly, there is growing evidence for CNVs associated with production traits and environmental adaptation. For example, in Holsteins cattle, 34 CNVs on 22 chromosomes have been characterized as being significantly associated with milk production traits, some of which are located within or near known QTL for milk production traits [21]. CNVs of the relaxin/insulin-like family peptide receptor 4 gene and the olfactory receptor are strongly associated with residual feed intake in Holstein cattle [22], whereas the CNV region of glycerol-3-phosphate acyltransferase 2 gene shows associations with carcass length and fatty acid composition in backfat and intramuscular fat [23]. Furthermore, the agouti signaling protein gene CNV allele has been shown to be almost entirely associated with coat color in Tibetan sheep [24]. 9805 CNV regions (CNVRs) are estimated to cover approximately 13.05% of the cattle genome and overlap with 5495 genes that are involved in environmental adaptation of Nellore cattle to tropical areas [25]. Apolipophorin 3 and fatty acid-binding protein 2, two genes involved in lipid transport and metabolism, are highly duplicated in the beef breeds [26]. These findings indicate that multiple beneficial CNVs may have been naturally selected in livestock during adaptation to different environments and could be associated with population diversity and economic characteristics.

The molecular mechanism underlying hypoxic adaptation in highland-local-animal has aroused attention for biological and medical research, not only because of the evolutionary significance of high-altitude adaptation, but also to understand human hypoxia-related diseases (e.g., acute mountain sickness, high-altitude pulmonary edema, and high-altitude cerebral edema [27]). For animal migrating to or living in high-altitude regions, environmental hypoxia is a primary challenge. The yak (*Bos grunniens*), a ruminant that separated from other ruminants approximately 2.2 million years ago, is mainly distributed in Qinghai-Tibetan Plateau (QTP) at altitudes ranging from 2500 to 6000 m, a region characterized by no absolutely frost-free period. It is known that yak is the only major livestock animal that can survive the extremely harsh environments of QTP, and are noted for their ability to tolerate the cold and anoxic conditions and resist the local disease threats. At present, there are more than 16 million domestic yaks, which provide the necessities for Tibetans and other nomadic pastoralists in high-altitude environments. In addition, there are 18 affirmed populations of the species in China, including the artificially breed Datong yak. It should be noted that the domestic yak is the only large animal that still coexists with its wild ancestors in similar environments [28]. Therefore, the yak provides a good framework for studying effects of CNV in hypoxic adaptation in large livestock. In previous studies, 161 CNV regions were detected from two yak individuals using the cattle-specific Nimblegen3x720K comparative genomic hybridization (CGH) array, and on the basis of a comparison between domestic and wild yak populations, Zhang et al. identified 121 potentially selected CNV regions harboring genes related to neuronal development, reproduction, nutrition and energy metabolism [29].

To date, few studies have confirmed the genome-wide presence of CNVs in relation to hypoxic adaptation in yak. Here, we selected 16 yak populations from different altitudes to conduct a genome-wide CNV analysis, and subsequently performed cluster analysis at both individual and population level. Initially, we conducted genome-wide CNV screening of the 16 yak populations, and thereafter performed GO enrichment analysis of CNVR-harbored genes to identify their functions. The information gained in this study will constitute a valuable resource for different yak populations for future studies on phenotypic variation and breeding, and will also provide important insights into the mechanisms underlying yak genomic evolution.

## Methods

### Tissue samples

Forty-eight healthy four-year-old yaks of similar weight were selected from 16 populations inhabiting widely distributed locations across the QTP (Additional file 1). The 16 studied yak populations were as follows: Dingqing (DQ), Shenzha (SZ), Gongbujiangda (GD), Cuona (CN), Jinchuan (JC), Zhongdian (ZD), Sibu (SB), Leiwuqi (LWQ), Pali (PL), Maiwa (MW), Gannan (GN), Jiulong (JL), Tianzhu (TZ), Datong (DT), Bazhou (BZ), Jiali (JL1). It should be noted that the Datong yak is an artificial breed, the founding male parent of which was a wild yak. For each population, triangular skin biopsy samples (ear notches) measuring approximately 15 mm were obtained from three individuals by using a pig ear notcher, Hemostasis at the biopsy site was accomplished by applying a spring paper clip over cotton gauze for 5 min [30]. The samples were immediately snap-frozen in liquid nitrogen.

### Construction of sequencing library, sequencing, and data processing

Genomic DNA was extracted and purified following the standard phenol/chloroform extraction procedure [31], and subsequently quantified using an Aliglent 2100 bioanalyzer (Agilent Technologies, Palo Alto, CA) and agarose gel electrophoresis [29, 32]. Paired-end sequencing libraries with an insert size of 500 bp were sequenced using an Illumian Hiseq 2000 platform.

Low-quality reads were filtered out using PRINSEQ (version 0.20.4) to obtain the clean reads in accordance with previously reported criteria [33]. Briefly, the following reads were removed: (1) reads with≧10% unidentified nucleotides; (2) reads for which more than 60% of the read length had a Phred quality value≦7; (3) reads with more than 10 bp aligned to the adapter; (4) duplicate reads; (5) reads with three consecutive base pairs for which Phred value was lower than 14; (6) reads shorter than 45 bp. The clean reads were mapped to the yak reference genome (BosGru_v2.0) using BWA-MEM (0.7.15-r1140) with set default parameters, and then the SAM format results were sorted and indexed into Binary Alignment Map (BAM) format files using SAM tools. Finally, Genome Analysis Toolkit (version 4.0.10.1) was used to realignreads located in regions around indels to reduce the inaccurate alignment.

### CNV identification and CNVR determination

The software CNVnator (v0.3.2), which is better adapted than other similar softwares with respect to the accuracy of the copy number estimation [34], was used for realigned BAM file processing. The parameters were selected following the recommendations of the author. After setting the sliding window to a fixed value of 100 bp, the following steps were run to calculate the number of reads in the interior and both sides of the window: CNVnator–tree, CNVnator–his, CNVnator–stat, CNVnator–partition, and CNVnator-cal. Thereafter, comparison of the average depth of re-sequencing data and the reference genome was used for further correction, thereby identifying the occurrence of CNV in the preset window.

To avoid bias caused by different coverage, the existence of undefined nucleotide (N) in the genome sequence, and false positives results of the CNVnator software, the CNVs obtained from previous steps were used for subsequent analysis. The average coverage depth for each sample and CNV, and the ratio of non-N bases for each CNV were calculated. For the deletion type of CNVs, the CNVs were selected as clean CNVs, which should satisfy the following conditions: (1) a ratio of non-N bases greater than 0.4 and the covered bases more than 50% of non-N bases; (2) an average coverage depth less than 60% of that in the sample. Condition for selection of the duplication type of CNVs were as follows: (1) a ratio of non-N bases greater than 0.9, with covered bases representing more than 90% of non-N bases; (2) an average coverage depth greater than 140% of that in the sample. Those CNVs satisfying the respective two conditions were selected as clean CNVs. In addition, we only retained the CNVs longer than 1.5 kb for further analysis.

The CNV region is defined as a combined region of overlapping CNVs on the yak genome. CNVRs are merged from different individuals with any amount of overlap by extending the boundaries of the overlapping CNVs. Here, *Perl script* was used for defining the CNVRs. Only CNVRs present in more than three individuals were used for subsequent functional and comparative analysis.

### Quantitative PCR (qPCR) and resequencing data validation

Quantitative PCR analysis was performed to validate the accuracy of the CNV assignments. 12 CNVRs that encompassed functional genes were selected for validation in 24 randomly-selected individuals. The bovine basic transcription factor 3 (*BTF3)* gene, for which no CNVs or segmental duplications (SDs) were detected in our analysis and in previous studies [29, 33], was selected as a reference location for qPCR validation. The M-Value and V-value for *BTF3* were 0.25 and 0.11, respectively, and thus the gene is considered to be very stable, and the normalization factor is reliable according to the thresholds suggested by Vandesompele et al. (i.e., ≤1.5 for M-value and ≤ 0.15 for V-value) [35]. The primers used for qPCR amplification were designed using Primer Premier 5.0 (Premier, Canada) software and synthesized by Invitrogen (Shanghai, China). These primers are listed in Additional file 2.

Real time qPCR assays were performed using SYBR Premix Ex Taq II(Perfect Real Time, Takara, Japan) according to the manufactures’ instructions. Gene expression was normalized to that of the reference *BTF3* gene. All real-time reactions, including controls with no templates, were carried out using a Bio-Rad CFX96 real-time PCR detection system (Bio-Rad, USA). Relative expression was calculated using the 2^-ΔΔCt^ method. Mean expression levels and standard deviations were obtained from three independent experiments.

### SDs detection and association with the distribution of CNV

Using yak BosGru_v2.0 genome assembly, a whole-genome assembly comparison approach was applied to detect putative SDs. Briefly, sequence identified as SDs should fulfil the conditions that the sequence is larger than 1 kb in length and has identity greater than 90%. The overlap between the SDs and CNVR was calculated using custom Perl script. Chi-square analysis of SD distribution in the genome and in CNVRs was then performed using the Chi.test package in R (version 3.3.1). In addition, using previously published *Perl script* [36], the association between CNVs and SDs was examined via random simulations.

### Gene annotation and ontology

To assess the gene in each CNVR, the coordinates of each CNVR in the yak genome assembly were determined and gene annotation was performed. For this analysis, we used those genes comprising more than 50% CNVR. Gene ontology (GO) enrichment analysis was performed using the online tool DAVID (https://david.ncifcrf.gov/). *P* values were adjusted by false-discovery rate (FDR). GO terms associated with CNVRs and whole genome background were plotted using WEGO online software [37] (http://wego.genomics.org.cn/).

### Heatmap analysis

Heatmap analysis was performed based on the sequencing depth obtained for each individual. Using the “depth” command in SAM TOOLS, the depth of each base was computed for each sample. The ratio of the average depth of each window to the effective depth for each individual was calculated as the estimated copy number. The estimated copy number values for all samples were then plotted using the heatmap function in the R package.

### Cluster analysis between different populations based on CNV

To identify CNV genes with high differentiation among the 16 populations surveyed in this study, the status of each CNV in each sample was defined as follows: deletion-type CNVs were designated − 1 and the amplification-type CNVs were designated 1. If no CNV were detected, this condition was designated 0. Using these values, we constructed a CNV matrix for each sample for clustering. Subsequently, we integrated the values obtained for three individuals in each population, and reconstructed a matrix for cluster analysis among populations. The metric formula used in this analysis has been reported previously [33].

### Statistics

The R package q-value (version 3.3.1) was used to calculate the FDR, and threshold for significant associations was set at a q-value of <0.05.

## Results

### Genome-wide identification of CNVs

A total of 0.98 Tbp sequences with an average depth of 8.1 × was obtained from the 48 individuals examined (Additional file 1). Aligning these sequences to the yak reference genome revealed that reads from single individual covered at least 67.5% of the genome and, on average, 75.69% of the reference genome was covered, indicating that the data are sufficient and of sufficiently high quality for CNV detection.

**Fig. 1:**
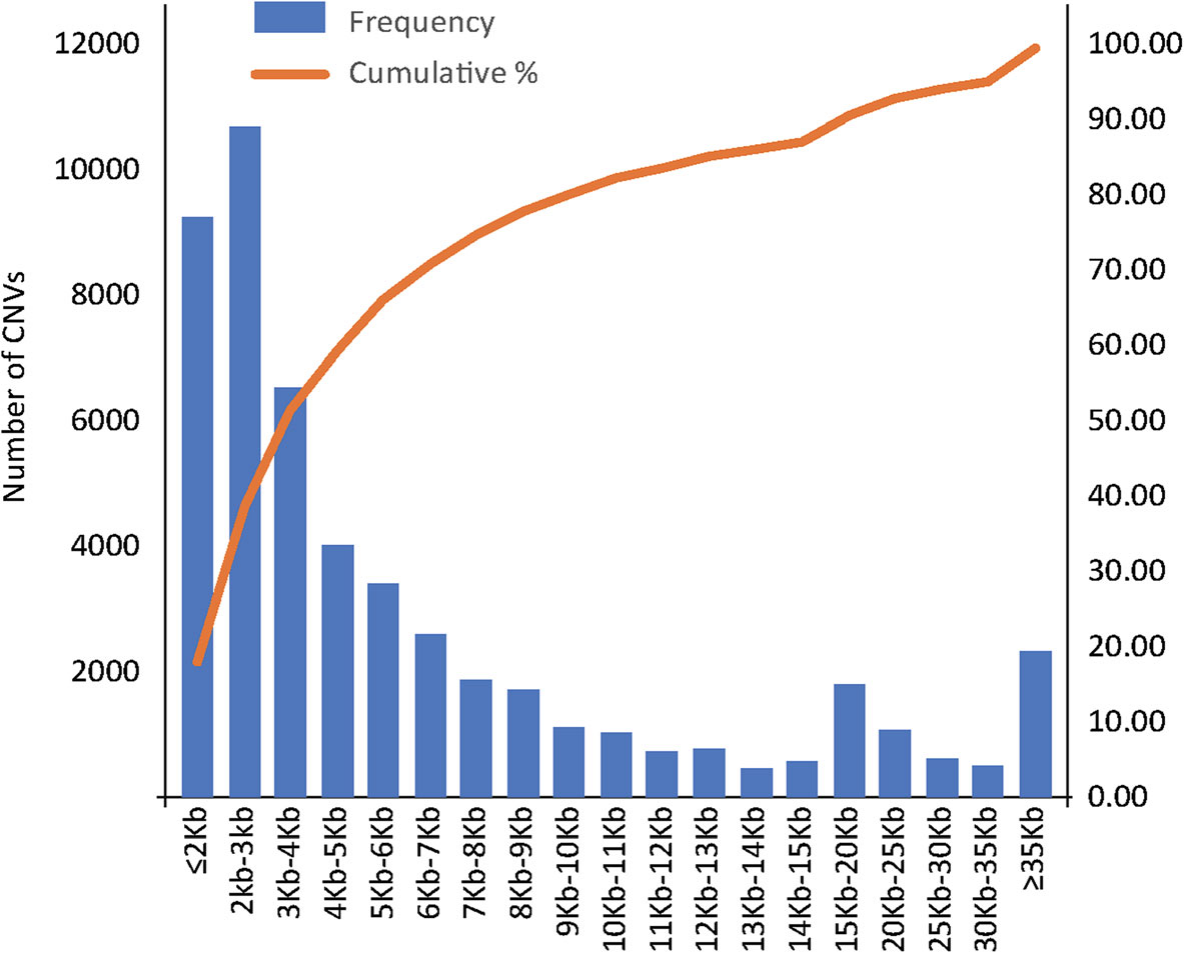
CNV size interval distribution, the average CNV size is 12.26 kb

The SNP distribution of the 48 individuals was obtained via comparing the detected genotype to polymorphism sites in the reference sequence. In total, we detected 247,811,300 SNP events among the 48 individuals, with an average number of 5,162,735 SNPs per individual (Additional file 3). Using CNVnator software based on the RD method [34], we detected a total of 51,461 CNV events (with an average of 24,729 gain and 26,732 loss events) from the 48 individuals (Fig. 1, Additional file 4), the average number of CNVs per individual was 1072 with an average of 557 gain and 515 loss events, and the average number of specific CNV events per individual was 107. The size of the CNVs identified varied from 1.5 kb to 1460.6 kb, with an average size of 12.26 kb. Details of the identified CNVs and location information for each individual are listed in Additional file 5. In addition, we found that CNVs were distributed in a non-random way and their contents vary across chromosomes.

**Fig. 2:**
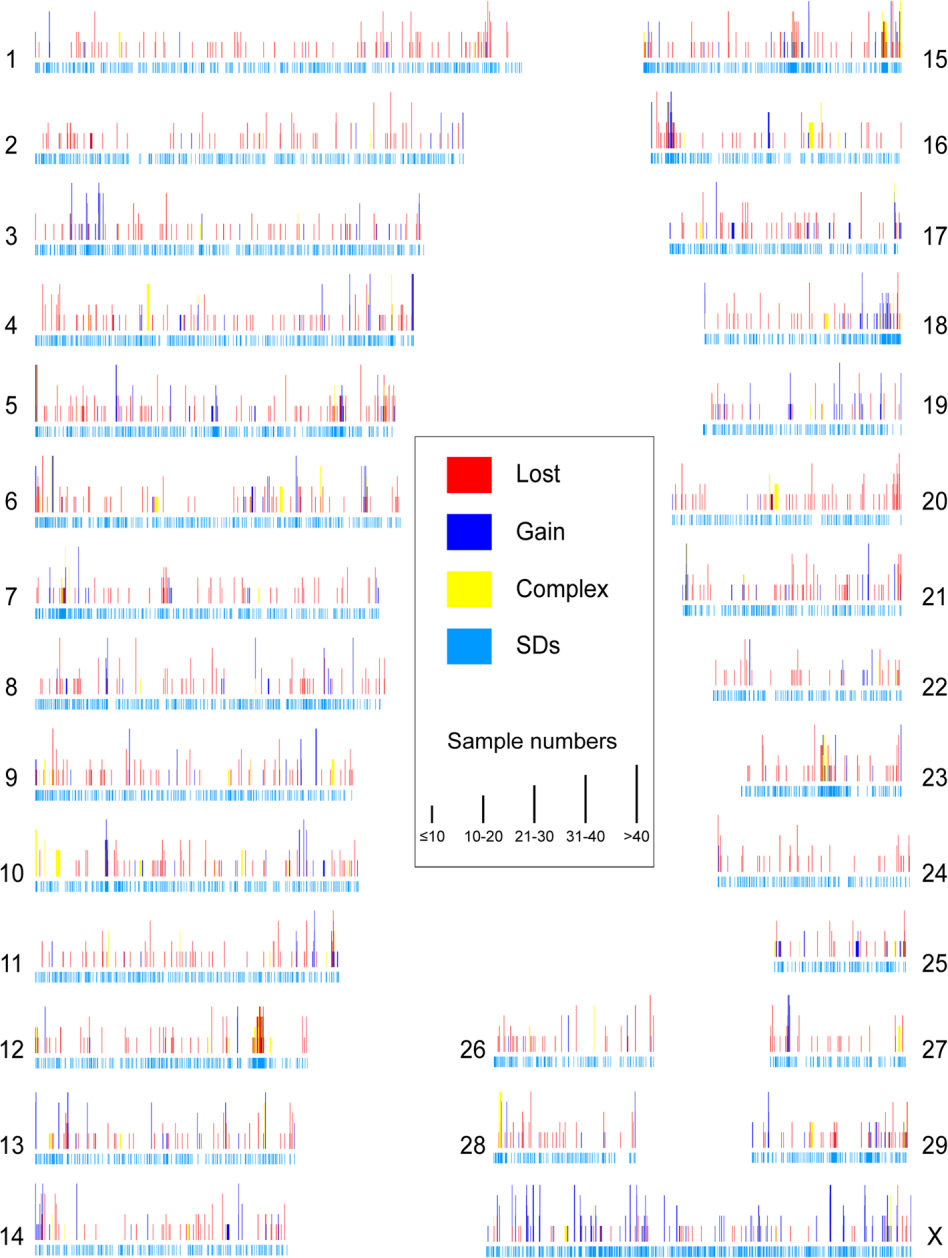
Genomic landscape of CNVRs and SDs in yak. The SDs are plotted as light blue bars. The CNVRs are illustrated above the SDs in rad (loss), navy blue (gain) and yellow (complex of gains and losses). The chromosome numbers (1–29, X) are shown nearby correspond CNV landscape, and the bar height represents different sample numbers (≤10、10–20、21–30、31–40、 > 40)

A total of 3154 CNVRs were defined by merging all overlapping calls across multiple individuals into unique regions and filtering out those that were present in fewer than three individuals (Additional file 6). These CNVRs occupied 163.8 Mb or 6.2% of the yak genome (Fig. 2). Furthermore, 28 CNVRs were found to be common to all 48 individuals. The detected CNVRs were divided into three categories according type: 1077 gain, 1776 loss, and 301 gain and loss (Additional file 6).

qPCR analysis was performed to evaluate the accuracy of individual CNVs predicted [38]. We accordingly found that 12 CNVRs overlapped functional genes and 24 different individuals were randomly selected for validation. The results showed that 89% of the CNVs (32/36) had an accurate copy number (Additional file 2).

### SDs detection and comparison with CNVRs

On the basis of whole-genome assembly comparison (WGAC) methods [39], we initially identified 27,705 putative SD events (Table 1). In this regard, it is interesting to note that of the 163.8 Mb CNVRs distributed across the yak genome, 10.8 Mb of high-confidence CNVRs directly overlapped with 52.9 Mb SDs.

**Table 1:** The summary of SDs in yak

Length (bp)	Events	Average length (bp)	Coverage (Mp)
1000	26,945	1615	45.199657
5000	718	5990	6.827168
10,000	42	11,097	0.875054

### Functional analysis of CNV-harbored genes

**Fig. 3:**
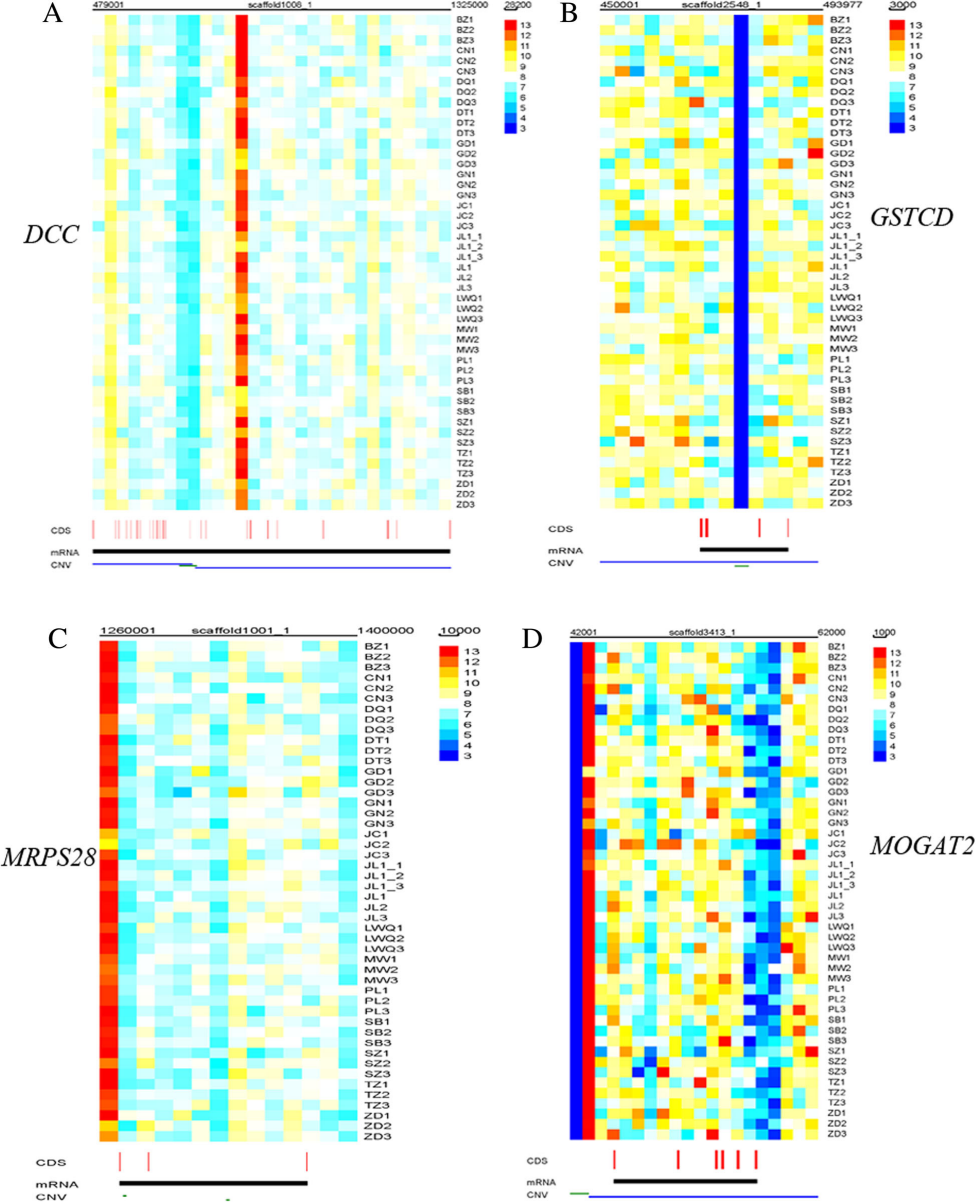
Heatmap of CNVR harbored genes. The heatmap of CNVR with *DCC* gene (**a**) located in scaffold1008_1, *GSTCD* gene (**b**) located in scaffold2548_1, *MRPS28* gene (**c**) located in scaffold1001_1, and *MOGAT2* gene (**d**) located in scaffold3413_1. The per bp of the genome that average read depths plotted is present in the top right corner for each CNVR harbored gene. CDS and gene are shown at the bottom, red line mean the coding sequence, and the black line mean the whole mRNA of gene

In total, 1374 protein-coding genes within or partially encompassed by the 3154 identified CNVRs were retrieved from the current yak genome (Additional file 7). In order to obtain insight into the biological functions of the CNVR-harbored genes, GO enrichment was performed using the DAVID bioinformatics resource. GO analysis annotated 765 of the 1374 genes in three main GO categories: molecular function, cellular component and biological process (Table 2). Genes in all categories were mainly involved in 23 processes, including transmembrane transporter activity [e.g., deleted in colorectal cancer (*DCC*)], guanyl ribonucleotide binding [mitochondrial ribosomal protein s28 (*MRPS28*)], purine metabolism, glucose metabolism [e.g., mono-acylglycerol O-acyltransferase 2 (*MOGAT2*)], immune response [e.g., dexi homolog (*DEXI*), class II major histocompatibility complex transactivator (*CIITA*), and SET and MYND domain containing 1(*SMYD1*)], sensory perception of chemical stimulus (e.g., dopamine receptor D3), and sensory perception (e.g., solute carrier family 26 member 4 gene). This set of CNV-harbored genes has a wide spectrum of molecular functions, cellular components and biological processes, and provides a rich resource for validating hypotheses on the genetic basis of phenotypic variation within and among different yak populations. Furthermore, it is worth noting that a series of CNVR-harbored genes that are associated with adaptation to high altitude [e.g., *DCC*, glutathione S-transferase C-terminal domain containing (*GSTCD*), *MRPS28*, and *MOGAT2*] showed significant differences in copy number among different populations living at different altitudes (Fig. 3).

**Table 2:** The significant GO categories of CNVR-harbored genes

GO ID	Function	GO type	Adjusted *p*-value	Number of CNV harbored genes	Number of all yak genes
GO:0015399	primary active transmembrane transporter activity	molecular function	0.000469	11	50
GO:0019001	guanyl nucleotide binding	molecular function	0.017441	33	328
GO:0032561	guanyl ribonucleotide binding	molecular function	0.024169	31	312
GO:0016887	ATPase activity	molecular function	0.038654	20	191
GO:0004871	signal transducer activity	molecular function	0.087474	53	640
GO:0060089	molecular transducer activity	molecular function	0.087474	53	640
GO:0006955	immune response	biological process	0.000127	19	108
GO:0002376	immune system process	biological process	0.000184	19	111
GO:0009259	ribonucleotide metabolic process	biological process	0.009166	16	122
GO:0009150	purine ribonucleotide metabolic process	biological process	0.017198	15	120
GO:0072521	purine-containing compound metabolic process	biological process	0.025628	15	126
GO:0009117	nucleotide metabolic process	biological process	0.026075	18	160
GO:0009119	ribonucleoside metabolic process	biological process	0.033152	14	119
GO:1901135	carbohydrate derivative metabolic process	biological process	0.035667	25	249
GO:0006753	nucleoside phosphate metabolic process	biological process	0.043746	18	170
GO:0007186	G-protein coupled receptor signaling pathway	biological process	0.047943	49	561
GO:0007606	sensory perception of chemical stimulus	biological process	0.049864	125	809
GO:0009116	nucleoside metabolic process	biological process	0.058703	14	129
GO:1901657	glycosyl compound metabolic process	biological process	0.058703	14	129
GO:0019637	organophosphate metabolic process	biological process	0.072145	21	218
GO:0055086	nucleobase-containing small molecule metabolic process	biological process	0.084247	18	185
GO:0007600	sensory perception	biological process	0.0031005	154	1023
GO:0071944	cell periphery	cellular component	0.024498	10	72

### Cluster analysis among individuals based on CNV

**Fig. 4:**
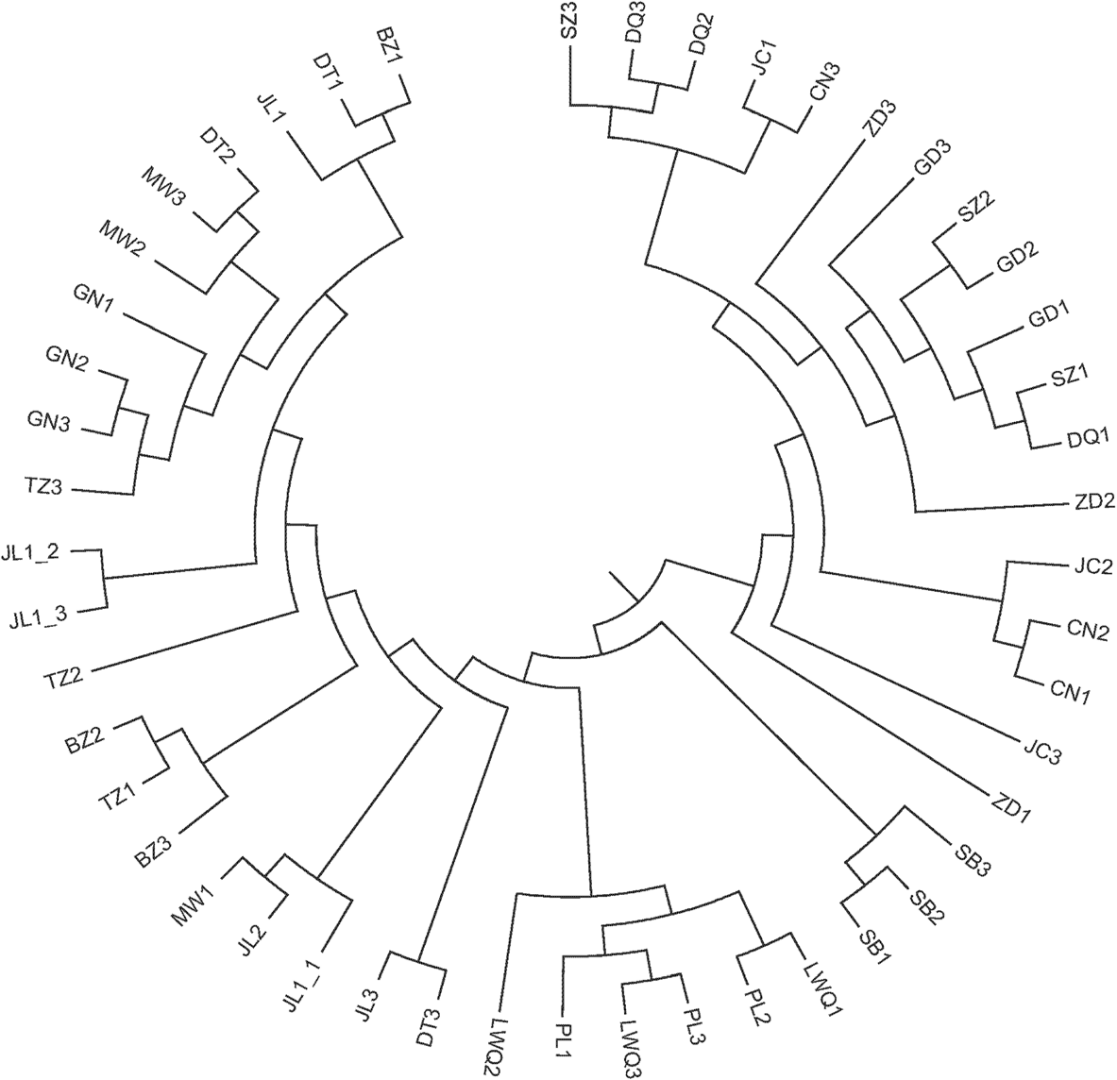
The clustering map in 48 individuals based on CNVs

**Fig. 5:**
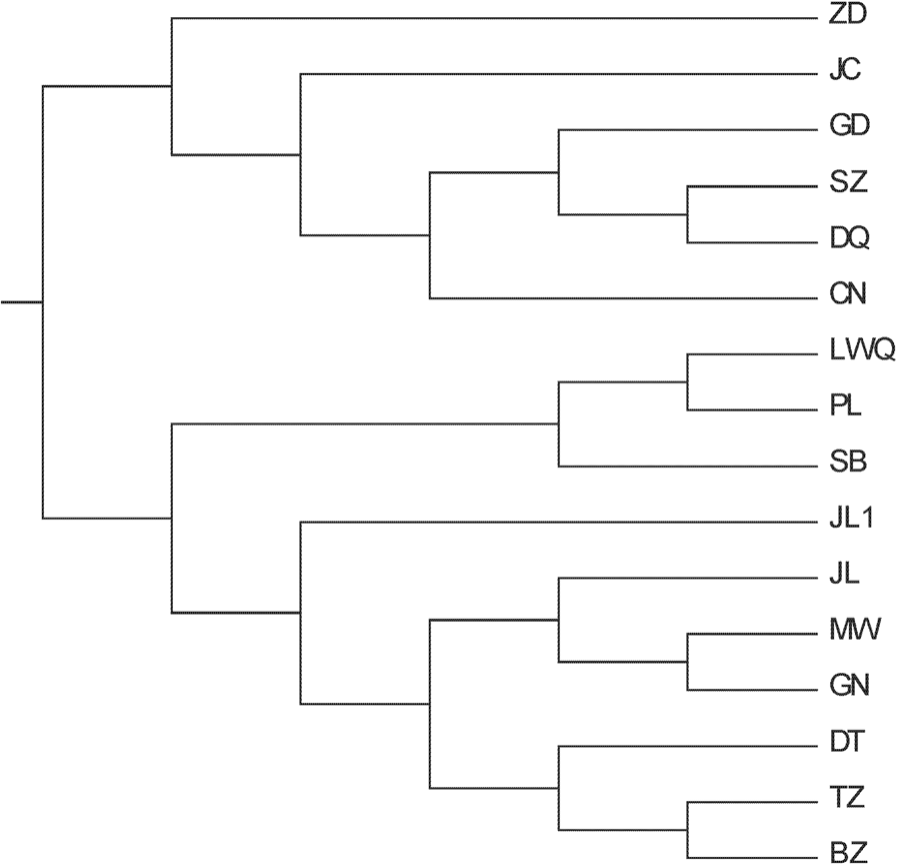
The clustering map in 16 populations based on CNVs

Using 48 selected yaks, we determined the different distribution events of CNVs in which we designated a copy number deletion event as − 1, an amplification event as 1, and a non-CNV as 0, and subsequently performed cluster analyze among the different individuals (Additional file 6). The results showed that the CNV clustering was divided into two branches (Fig. 4). Furthermore, cluster analyses were performed based on 16 populations (Fig. 5), and the results further indicated that the 16 populations were consistently classified into two continental groups.

## Discussion

In this study, we detected and verified CNVRs using whole-genome resequencing and qPCR, the results showed that the CNVRs occupied 163.8 Mb or 6.2% of the yak genome, which is slightly higher than the value determined in previous study that examined 14 wild and 65 domestic yaks (153 Mb, 5.7%) via resequencing [33] and considerably higher that than determined for two yak individuals (33 Mb, 1.25%) based on a CGH array approach [29]. Due to differences in the technology employed and the individual samples used for CNVs analysis, even though we found that the number trend of the three categories was: loss CNVRs > gain CNVRs > gain and loss CNVRs, it is difficult to compare the CNVs detected in different studies. In the present study, using next-generation sequencing, we achieved greater confidence and better resolution in calling CNVs than has been obtained previously. Therefore, compared with previous studies, most of these newly discovered CNVRs are novel, and thus further supplement the research base of CNVs in yak. In addition, for the first time, our study focuses on the genome CNV maps of different yak populations.

The validation of the accuracy of individual CNVs by qPCR showed that 89% of the CNVs had an accurate copy number. It should be note, however, that, given the complex structure of CNVRs and low-copy duplications with lower sequence similarities, false positive identification is common in CNV detection via qPCR analysis [29, 40]. Furthermore, the quality of the assembled reference and the annotated repeats plays a key role in discovering CNVs using the RD method [26]. Consequently, in order to preclude false positives from analysis, fluorescence in situ hybridization and array comparative genomic hybridization [41, 42] will be required to obtain more accurate information in further studies.

Taking into consideration that segmental duplications (SDs) are among the major catalysts and hotspots for CNV formation in mammals [43], we sought to determine whether there are association between the CNVs and SDs in yak. After analysis, we found that 10.8 Mb of the 163.8 Mb CNVRs directly overlapped with 52.9 Mb SDs. We speculate that the SDs distribution pattern is predominant in yak CNVs, which is consistent with previous studies showing that CNVs are enriched with SDs [44].

Our finding that the CNV-harbored genes are enriched in sensory perception is consistent with the findings of previous studies, which have shown that there are a large family of olfactory genes and that these are associated with CNVs in humans [45], mice, and dogs [46]. This apparent conservation of CNVs across mammalian species may be attributable, in part at least, to the fact that selective pressure might drive the acquisition or retention of specific gene dosage alterations, and that the gene families involved in sensory perception are typically rapidly evolving because they play key roles in the response of organisms to rapid changes in the environment and have been repeatedly detected in CNVRs of cattle, mouse, and dog genomes [47, 48]. The functional category enrichment of CNV-harbored genes may be a reflection of their physiological role in the regulation of hypoxic adaption, species evolution and biodiversity.

The DCC gene encodes a netrin receptor, a key regulator in DCC/APPL-1/AKT pathway, which attenuates hypoxia-induced neuronal apoptosis and improves neurological function [49, 50]. GSTCD, a member of a subgroup of the Glutathione S-Transferase (GST) gene family, plays a specific role in protecting against the products of oxidation stress, and its expression is induced by compounds associated with chemical stress and carcinogenesis [51]. MRPS28 is generally expressed in oxygen sensitive organs, including the brain, cerebellum, and kidney. Mitochondria are the primary energy-generating system in multiple eukaryotic cells, and are the energy supply centers for cellular processes, such as intermediary metabolism, calcium signaling, and apoptosis. MOGAT2, a member of MOGAT gene family, plays an important role in catalyzing the metabolism of triglycerides and is highly conserved in organisms. In this study, *MOGAT2* was found to have different degrees of copy number amplification and deletion in the 16 yak populations. Heat map analysis also revealed that there is a significant difference in the CNV of the *MOGAT2* among these individuals, which indicates that the mechanisms of fat metabolism and carbohydrate utilization are important to yak production and reproductive performance in the severe environment of the Qinghai-Tibetan Plateau. Furthermore, we found *DEXI* [52], *CIITA* [53], and *SMYD1* [54] are key genes influencing the immune system and may reflect the substantially different diseases triggered by the parasites and arbovirus found at high altitudes. These results suggest that CNV is a key type of genetic variation that may play an important role in yak adaptation to high-altitude environments, and thus it is desirable to carry out further research on the specific relationships or interaction mechanisms between the function of validated genes and CNV.

Our CNV clustering results based on individuals is consistent with the theory that the yaks have two different ancestral origins [55], although we also found that individuals within a single population showed cross clustering, only SB yaks were found to cluster together and at a distance from other yak populations, thereby indicating that CNVs may play an important role in the adaptation to hypoxia in SB yaks and their unique characters. In contrast to SNP-based cluster analysis, different yaks in the same population were found to cluster further from each other in clustering based on CNVs. For instance, two individuals from the LWQ yak population were clustered with the PL yak population, whereas the other individual in LWQ formed a solitary group. These findings are consistent with previous studies that have indicated that the characteristics of CNV-based clustering can show large variations between individuals, and that introgression plays a potentially important role in yak adaptation [56]. Indeed, cluster analysis of CNV variation has geographic distribution characteristics. For instance, TZ, DT, GN and MW and JL yaks are geographically close, which consequently enhances the opportunities for exchange genetic information among these populations, and is reflected in their closer clustering based on the coefficient of CNV. Similar results were observed for LWQ, SB and PL1 yaks. On the basis of these differences of CNV frequency among yaks, we hypothesize that yak CNVs are likely to arise independently in individuals/populations and contribute to individual/population differences and therefore are related to the population formation and adaptation, and gene communication exist between some populations. Different depths of sequence would markedly affect the detection rate and feature evaluation of CNV. To eliminate this imbalance as much as possible, cluster analyses were further performed based on populations, and the results indicated that the yak populations involved in this study were consistently classified into two continental groups.

## Conclusions

In this study, we employed whole-genome sequencing to investigate the genome-wide CNV for 48 yaks in 16 populations. A total of 24,729 gain and 26,732 loss events, and 3174 CNVRs covering 163.8 M (6.2%) of the yak genome were identified. These CNVs provide the largest source of these variations identified to date along with the highest-resolution CNVR and SD distribution maps for the 30 chromosomes of yak. The potential CNVRs contain 1374 functionally annotated genes and GO enrichment analysis revealed that these CNVR-harbored genes are largely related to oxidative phosphorylation, immune response, olfactory receptor activity, and sensory perception. Some novel CNVR-harbored genes, including *DCC*, *GSTCD*, *MRPS28* and *MOGAT2*, are probably associated with the adaptation to high-altitude environments. In addition, the findings of our study support the hypothesis that the yak populations are mainly composed of two distinct ancestries. Taken together, our results constitute a valuable genome-wide variation resource of different yak populations for future work on phenotypic variation and breeding in yaks and provide insights into the mechanisms underlying yak genome evolution.

## Additional files

Additional file 1: The basic information relating to the yak used in present study and the resequencing data used in the CNV analysis. (XLSX 17 kb)

Additional file 2: The primer sequences for qPCR and the confirmation results. (XLSX 13 kb)

Additional file 3: Genome-wide SNP diversity in 48 individuals. (XLSX 14 kb)

Additional file 4: The summary of CNV events in each individual. (XLSX 15 kb)

Additional file 5: List of all CNVRs in the yak genome. (XLS 3420 kb)

Additional file 6: List of the potential 3154 CNVRs. (XLS 1175 kb)

Additional file 7: The protein-coding genes within or partially inside of the identified 3154 CNVRs. (XLS 226 kb)

## Abbreviations

*BTF3*: Basic transcription factor 3
BZ: Bazhou yak
CGH: Comparative genomic hybridization
CN: Cuona yak
CNVR: Copy number variation region
CNVs: Copy number variations
*DCC*: Deleted in colorectal cancer
DQ: Dingqing yak
DT: Datong yak
FISH: Fluorescent in situ hybridization
GD: Gongbujiangda yak
GN: Gannan yak
GO: gene ontology
*GSTCD*: glutathione S-transferase C-terminal domain containing
JC: Jinchuan yak
JL: Jiulong yak
JL1: Jiali yak
LWQ: Leiwuqi yak
*MOGAT2*: mono-acylglycerol O-acyltransferase 2
*MRPS28*: mitochondrial ribosomal protein s28
MW: Maiwa yak
PL: Pali yak
qPCR: quantitative PCR
SB: Sibu yak
SDs: segmental duplications
SZ: Shenzha yak
TZ: Tianzhu yak
ZD: Zhongdian yak
